# Leigh syndrome global patient registry: uniting patients and researchers worldwide

**DOI:** 10.1186/s13023-023-02886-0

**Published:** 2023-09-04

**Authors:** Sophia Zilber, Kasey Woleben, Simon C. Johnson, Carolina Fischinger Moura de Souza, Danielle Boyce, Kevin Freiert, Courtney Boggs, Souad Messahel, Melinda J. Burnworth, Titilola M. Afolabi, Saima Kayani

**Affiliations:** 1 Cure Mito Foundation, 6808 Old Glory Ct., McKinney, TX 75071 USA; 2 Faculty of Health and Life Sciences, Northumbria University, A521A Ellison Building, Newcastle Upon Tyne, NE1 8ST UK; 3 University of Washington, Seattle, WA USA; 4 Medical Genetic Service, Hospital de Clinicas de Porto Alegre, Rua Ramiro Barcelos, 2350, Porto Alegre, RS 90035-903 Brazil; 5 Biomedical Informatics and Data Science Section, Johns Hopkins University School of Medicine, 2024 East Monument St. S 1-200, Baltimore, MD 21205 USA; 6 Peter O’Donnell Jr. Brain Institute, University of Texas Southwestern Medical Center, 5323 Harry Hines Boulevard, J4.122, Dallas, TX 75390-8802 USA; 7 University of Texas Southwestern Medical Center, 5323 Harry Hines Blvd, Dallas, TX 75390 USA; 8 Department of Pharmacy Practice, Midwestern University College of Pharmacy, Glendale Campus, 19555 N. 59th Ave, Glendale, AZ 85308 USA; 9 Phoenix Children’s Hospital, Arizona, USA

**Keywords:** Leigh syndrome, Leigh disease, Mitochondrial disease, Patient registry, Patient driven, Real world data, Clinical trials, Rare disease, Research, Hope

## Abstract

**Background:**

Leigh Syndrome (LS) is a rare genetic neurometabolic disorder, that leads to the degeneration of the central nervous system and subsequently, early death. LS can be caused by over 80 mutations in mitochondrial or nuclear DNA. Patient registries are important for many reasons, such as studying the natural history of the disease, improving the quality of care, and understanding the healthcare burden. For rare diseases, patient registries are significantly important as patient numbers are small, and funding is limited. Cure Mito Foundation started a global patient registry for LS in September 2021 to identify and learn about the LS patient population, facilitate clinical trial recruitment, and unite international patients and researchers. Priorities were to allow researchers and industry partners to access data at no cost through a clear and transparent process, active patient engagement, and sharing of results back to the community.

**Results:**

Patient registry platform, survey design, data analysis process, and patient recruitment strategies are described. Reported results include demographics, diagnostic information, symptom history, loss of milestones, disease management, healthcare utilization, quality of life, and caregiver burden for 116 participants. Results show a high disease burden, but a relatively short time to diagnosis. Despite the challenges faced by families impacted by Leigh syndrome, participants, in general, are described as having a good quality of life and caregivers are overall resilient, while also reporting a significant amount of stress.

**Conclusion:**

This registry provides a straightforward, no-cost mechanism for data sharing and contacting patients for clinical trials or research participation, which is important given the recruitment challenges for clinical trials for rare diseases. This is the first publication to present results from a global patient registry for Leigh Syndrome, with details on a variety of patient-specific and caregiver outcomes reported for the first time. Additionally, this registry is the first for any mitochondrial disease with nearly 70% of participants residing outside of the United States. Future efforts include continued publication of results and further collaboration with patients, industry partners, and researchers.

## Introduction

Leigh syndrome is a rare genetic neurometabolic disorder, leading to the degeneration of the central nervous system and subsequently early death. Leigh syndrome is considered the most common type of rare pediatric mitochondrial disease affecting around 1 in 40,000 individuals [1, 2]. The onset of symptoms usually occurs between the ages of three months and two years, but some patients present with a later disease onset. Leigh syndrome can be caused by over 110 genetic mutations in mitochondrial or nuclear DNA [3].

A patient registry is an organized system that uses observational study methods to collect uniform data (clinical and other) to evaluate specified outcomes for a population defined by a particular disease, condition, or exposure, and that serves one or more predetermined scientific, clinical, or policy purposes [4].

For mitochondrial diseases, it is widely acknowledged that the rarity and heterogeneity of individual genetic diseases combined with the lack of patient registries to understand the natural history of each unique disease and enable designing rigorous clinical trials, contribute to the challenges researchers face in developing targeted therapies. To combat this, innovative partnership across the bioscience ecosystem centered on patients is required [5]. Patient registries are important to understand the natural history of the disease, measure and improve quality of care, and understand the healthcare burden [4]. More importantly in rare diseases, patient registries have become efficient tools in providing valuable patient data, considering the heterogeneous disease profile, small patient population, and limited funding [6, 7].

Cure Mito Foundation recognized these challenges and initiated the Leigh syndrome Global Registry in September 2021. Cure Mito Foundation is a parent-led, fully volunteer foundation dedicated to advancing education and research for Leigh syndrome and mitochondrial disease. The goals of the registry are to further describe Leigh syndrome clinical characteristics and phenotype, enable and facilitate clinical trials recruitment, and unite international patients and researchers. Mesko and colleagues share the viewpoint that a more active, collaborative process that engages with patients as true partners at all times optimizes the wants and priorities of the ultimate stakeholder, the patient. This process is referred to as patient-designed care [8].

To this end, patient foundations led by affected patients and their caregivers (parents) likely represent the true understanding of the impact of the disease on the patients’ immediate needs (real world data) and create a sense of urgency in driving research further. This is reflected in the design of the Leigh syndrome registry, which encourages active patient engagement, data availability at no cost to interested researchers and industry partners, and transparent sharing of findings and results with the wider community.

We believe there is an increasing need for better understanding of Leigh syndrome with an aim to support the development of transformative therapies, driven by the unmet need of treatment options for patients with this genetic disease. This paper is, to our knowledge, the first available publication on data from a global Leigh syndrome patient registry.

## Materials and methods

### Registry platform

Cure Mito Leigh syndrome Global Registry was initiated in partnership with Coordination of Rare Diseases at Sanford (CoRDS). CoRDS is a disease agnostic platform with data for over 2000 rare diseases. CoRDS registry platform was established in 2010, and Sanford Health was founded in 1894.

### Surveys design

The registry consisted of two surveys: general and Leigh syndrome specific. The general survey is provided by CoRDS and is the same for all rare diseases on the CoRDS platform and is using Common Data Elements (CDE) provided by the National Institutes of Health (NIH) [9]. The Leigh syndrome survey was developed by the Cure Mito Foundation with input from Matthew Klein, MD, CEO of PTC Therapeutics—the industry partner conducting clinical trials in mitochondrial disease—and Cure Mito medical and scientific advisory board members. For participants under 18 years old, a parent (or legal guardian) completed the survey. Adult participants who are cognitively impaired and unable to complete the survey by themselves, were also enrolled and their legal guardians completed the survey on their behalf. Surveys could be completed online, by email, mail, or phone. The word “participant” throughout the paper refers to an individual with Leigh syndrome.

### IRB approval and data access

Patient registry and surveys are ethically approved by the CoRDS Institutional Review Board and listed on ClinicalTrials.gov (NCT01793168). CoRDS provides a streamlined service for researchers or industry representatives, interested in facilitating clinical trial or research design or recruitment at no cost. Requests to either obtain data or share clinical trials or research opportunities with registry participants are evaluated by the CoRDS Scientific advisory board with input from Cure Mito Foundation. As CoRDS is a disease agnostic platform, it was selected in part based on its approach of being unbiased, with comparison to a more common setup, where a patient organization or a group of stakeholders with vested interest in the disease, having full oversight of data access.

### Informed consent

Participants or their caregivers provided informed consent to be contacted for future research studies, for the donation of biospecimens, and the sharing of de-identified data with researchers. Participants or their caregivers were also given the option to consent to sharing their data and contact information with the Cure Mito Foundation.

### Information collected

Information collected included: demographic, mitochondrial disease diagnosis, loss of milestones, disease management, specialists seen, history of symptoms, healthcare utilization and infections within three and 12 months, caregiver burden, and quality of life.

The caregiver burden part of the survey was Neuro-QoL Caregiver v2.0—TBI-CareQOL Caregiver Strain—Short Form 6a [10], which is a validated survey initially created for individuals with traumatic brain injury and used with the permission of the authors.

Participant’s genetic report can be uploaded through the registry platform.

Survey responders are contacted annually by the CoRDS registry with a reminder to update their information, however responders also have an opportunity to make updates at any time. This way, data can be collected longitudinally.

### Data cleaning

The surveys were designed to include branching logic to help reduce the number of data inconsistencies. The survey was also configured to allow uniformity in data reporting and presentation. Free text data entries such as genetic mutation information were re-coded to conform to a consistent format. Data values that were incorrect and data points that were incomplete were noted as "missing."

### Data analysis

Data was analyzed using SAS version 9.4. Descriptive statistics were used, including mean, standard deviation, mode, median, interquartile range, and range. Independent quality control checks of selected results were performed.

Cure Mito Foundation has a unique strength of having data analysis expertise within the organization, to support data and statistical analysis. This allowed for rapid and efficient analysis of the collected data and sharing of the results through posters and presentations at multiple conferences, as well as sharing results through graphics on social media.

### Participants recruitment

Participants or their caregivers were invited to join the registry through posts and videos on Cure Mito social media, Leigh syndrome Facebook groups and newsletters, with the help of Cure Mito partner families. Partner families are families affected by Leigh syndrome who join Cure Mito in support of its mission and are able to reach out to their own communities in their own language. For patients meeting eligibility criteria, informed consent was obtained, and the voluntary nature of participation was highlighted. Participating families were allowed to withdraw from the registry at any time.

In an effort to promote transparency and open dialogue with the community, Cure Mito Foundation shared on their website the purpose of the registry, what information is collected, how Cure Mito will use the data, and how data will be accessed and protected.

### Inclusion/exclusion

Participants who met the following eligibility were included in the analysis in this paper :

#### Inclusion criteria

Diagnosis of Leigh syndrome confirmed by gene mutation, as reported in the registry.

#### Exclusion criteria

Patients without confirmed mutations in genes associated with Leigh syndrome.

Detailed inclusion and exclusion criteria can be seen in Appendix, Table 6.

## Results

### Demographics

**Fig. 1:**
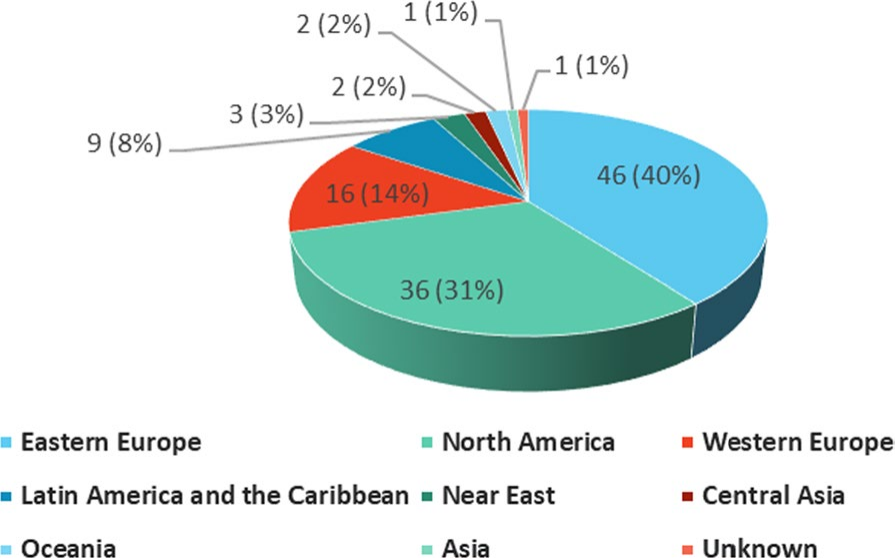
Geographic region (N = 116)

A total of 116 participants were included, with equal number of participants (47% each) were female and male (~7% did not report their biological sex), 78% of the participants were white, 91% were alive at the time of survey submission (Table 1). Mean age of living participants at survey submission in years was 7.1 (median 5.1, SD 9.3). Largest age category was 3–5 years old (37%, Appendix, Fig. 9). For deceased participants, mean age at death was 3.5 years (median 2, SD 4.3). The most represented geographic region was Eastern Europe (40%), followed by North America (31%) (Fig. 1). 25 countries were represented (Appendix, Table 7), among which the United States (US) was the most represented (31%), followed by Russia (28%). Only 2 participants completed the survey themselves, for the rest it was completed by a parent or legal representative.

**Table 1:** Participant Characteristics (N = 116)

Sex n (%)	
Female	54 (46.55)
Male	54 (46.55)
Not reported	8 (6.90)
Race n (%)	
White	90 (77.59)
Other/Unknown/Not reported	17 (14.66)
Asian–Indian	3 (2.59)
Pacific Islander–Other	2 (1.72)
American Indian or Alaska Native	1 (0.86)
Asian–Korean, White	1 (0.86)
Black or African American	1 (0.86)
Black or African American, White	1 (0.86)
Alive at the time of survey submission	
Yes	105 (90.52)
No	11 (9.48)
Survey responder	
Caregiver	114 (98.28)
Participant	2 (1.72)
Age at survey submission, years (Living participants only, N = 105)	
Mean (SD); Mode	7.1 (9.3); 4
Median (Q1, Q3); Min, Max	5.0 (3.0, 7.0); 0, 68
Age at death, years (deceased participants only, N = 11)	
Mean (SD); Mode	3.5 (4.3); 1
Median (Q1, Q3); Min, Max	2.0 (1.0, 4.0); 0, 14
Age at diagnosis, years	
Mean (SD); Mode	2.8 (5.0); 1
Median (Q1, Q3); Min, Max	1.8 (1.0, 2.8); 0, 46
Genome type n (%)	
nDNA	74 (63.79)
mtDNA	42 (36.21)
Specialists currently seen, Living participants only (N = 88)	
Mean (SD); Mode	5.7 (4.2); 6
Median (Q1, Q3); Min, Max	5.0 (3.0, 8.0); 1, 30

Abbreviations: nDNA = nuclear DNA; mtDNA = mitochondrial DNA

### Mitochondrial disease diagnosis

**Fig. 2:**
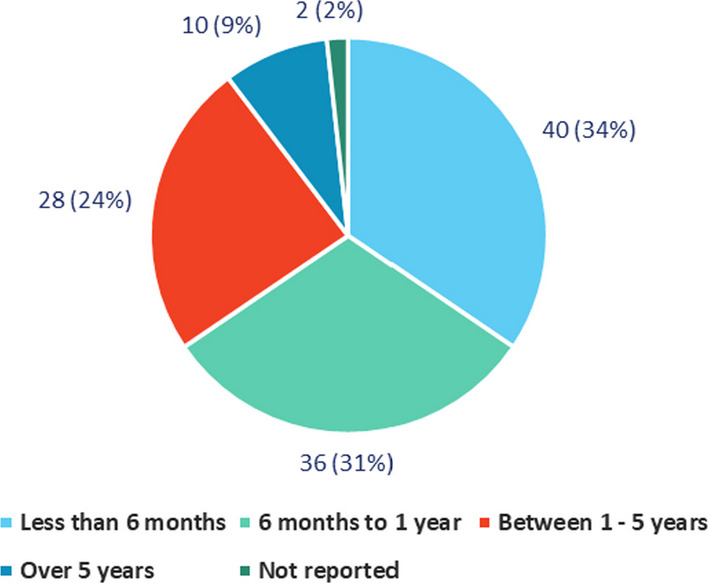
Time to diagnosis (N = 116)

**Fig. 3:**
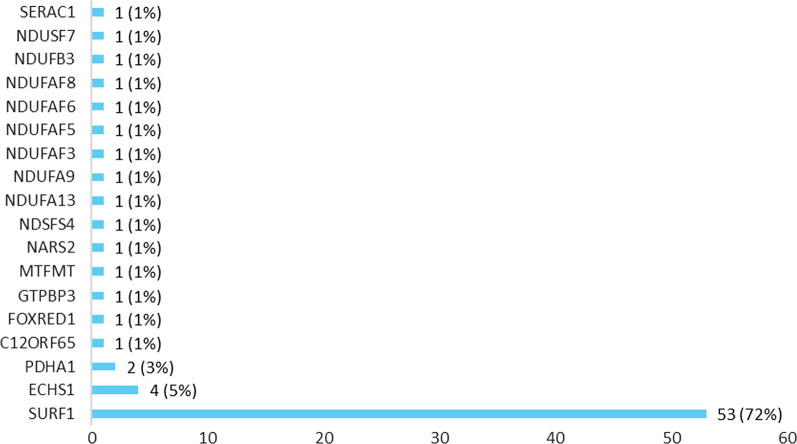
nDNA mutations (N = 74)

**Fig. 4:**
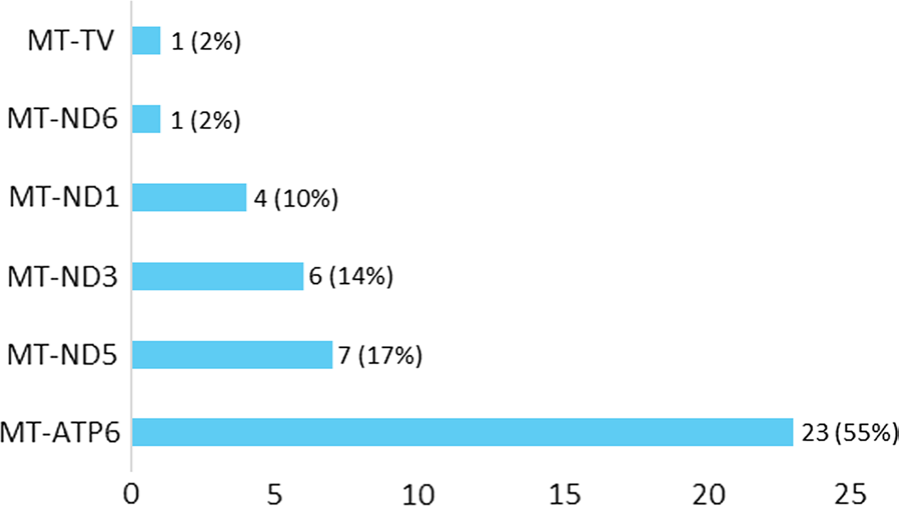
mtDNA mutations (N = 42)

Mean age (years) at diagnosis is 2.8 (Median 1.8, SD 5) (Table 1). For 64% of the participants, the diagnosis is caused by nuclear DNA mutation, and for the rest by mitochondrial DNA mutation. Among nuclear DNA genes, SURF1 was the most prevalent gene (72%), with 18 different nuclear DNA genes reported (Fig. 3). Among mitochondrial DNA genes, most prevalent was MT-ATP6 (55%), with 6 different mitochondrial DNA genes reported (Fig. 4). Types of genetic testing methods used to confirm diagnosis are shown in Appendix, Table 8. Time from disease onset to diagnosis is under 6 months for 34% of participants, and between 6 and 12 months for 31%. Only 9% of participants reported time to diagnosis as over 5 years (Fig. 2).

### First concerns reported and history of symptoms

**Fig. 5:**
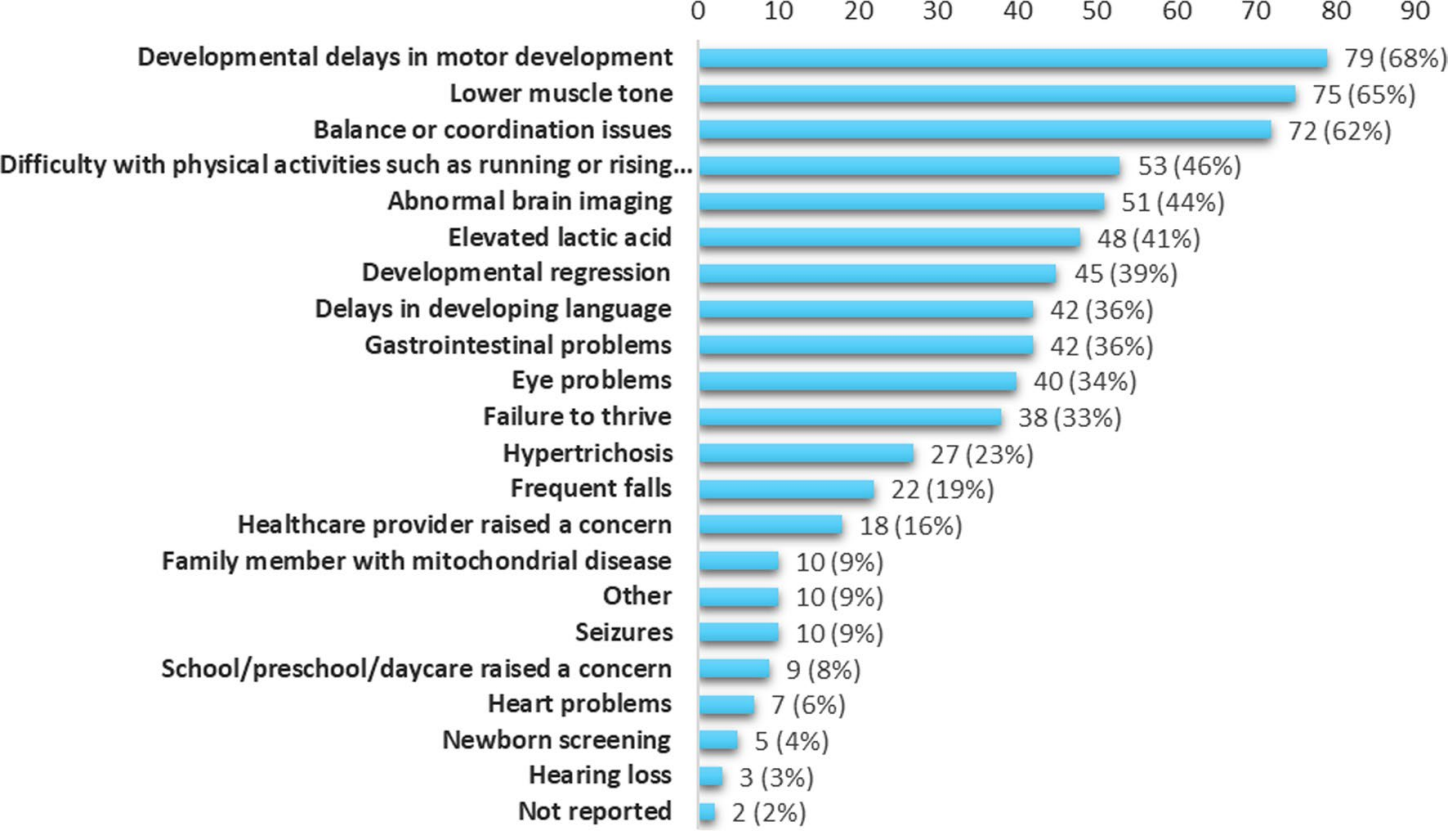
First concerns reported

Among participants' first symptoms reported, the most prevalent was developmental delay (68%), followed by lower muscle tone (65%), and balance and coordination issues (62%) (Fig. 5). Regarding history of symptoms overall, the most prevalent symptom was hypotonia (80%), followed by failure to thrive (71%) (Table 2).

**Table 2:** History of symptoms

			Age first experienced symptom (years)		
Symptom	N^a^	Symptom experienced	N^b^	Mean (SD); Mode	Median (Q1, Q3); Min, Max
		n (%)			
Hypotonia	100	80 (80.00)	47	1.2 (1.4); 1	1.0 (0.4, 1.5); 0, 9
Failure to thrive	97	69 (71.13)	55	1.3 (1.2); 1	1.0 (0.4, 2.0); 0, 6
Nystagmus or strabismus	105	69 (65.71)	52	2.2 (2.5); 1	1.5 (0.9, 2.3); 0, 15
Seizures	104	46 (44.23)	32	2.9 (4.1); 1	1.0 (0.4, 3.0); 0, 18
Gastric reflux	98	45 (45.92)	34	1.5 (1.7); 1	0.9 (0.3, 2.0); 0, 6
Hand tremor	97	44 (45.36)	35	2.9 (2.9); 2	2.0 (1.0, 3.0); 0, 16
Dystonia	98	40 (40.82)	30	3.3 (3.5); 2	2.0 (0.8, 3.0); 0, 16
Gastric motility issues	91	31 (34.07)	24	3.7 (6.5); 1	1.8 (1.0, 3.0); 0, 30
Sleep apnea	96	28 (29.17)	22	2.7 (1.7); 2	2.5 (1.7, 4.0); 0, 6
Tics	96	17 (17.71)	15	3.6 (2.3); 4	3.0 (2.0, 4.0); 0, 9

^a^N = number of participants with a valid response (Yes, No) regarding a specific symptom

^b^N = number of participants who had a history of a symptom and provided an age when it first started

### Specialists seen

Among living participants, the number of specialists currently seen ranged from 1 to 30 with mean 5.7 (median 5.0, SD 4.2) (Table 1). The most commonly seen specialists were the neurologists (78%); all categories can be seen in Appendix, Table 9. Other specialists seen included music therapist, hippo therapist, and visual therapist.

### Loss of milestones

The most affected developmental milestone was walking: 40% of participants never walked, 38% lost the ability to walk at a median age of 2 years (Table 3).

**Table 3:** Loss of milestones

					Age when milestone was lost (years)		
Milestone	N^a^	Lost	Never achieved	Lost or never achieved	N^b^	Mean (SD); Mode	Median (Q1, Q3); Min, Max
		n (%)	n (%)	n (%)			
Walk	112	42 (37.50)	44 (39.29)	86 (76.79)	33	3.5 (5.6); 2	2.0 (2.0, 3.0); 1, 33
Crawl	114	39 (34.21)	25 (21.93)	64 (56.14)	30	4.1 (6.5); 2	2.0 (1.4, 3.0); 1, 29
Sit up	114	40 (35.09)	18 (15.79)	58 (50.88)	34	3.1 (4.2); 2	2.0 (1.0, 3.0); 1, 24
Be toilet trained	113	19 (16.81)	35 (30.97)	54 (47.79)	16	3.5 (2.0); 2	3.0 (2.0, 5.0); 1, 8
Speak whole sentences	113	19 (16.81)	35 (30.97)	54 (47.79)	13	3.6 (2.4); 2	3.0 (2.0, 4.0); 1, 9
Speak 2-word combinations	98	25 (25.51)	24 (24.49)	49 (50.00)	17	2.9 (2.3); 2	2.0 (2.0, 3.0); 1, 9
Swallow	114	34 (29.82)	6 (5.26)	40 (35.09)	28	3.6 (3.6); 3	3.0 (1.1, 3.5); 0, 17
At least one milestone	116	71 (61,21)	65 (56.03)	97 (83.62)			

^a^N = number of participants with a valid response (Yes, No, Too young, Never achieved) regarding a specific milestone

^b^N = number of participants who lost a milestone and provided an age when the milestone was lost

### Disease management

The most prevalent type of intervention or adaptive devices used was mobility devices (72%) (Table 4). Types of mobility devices used included wheelchair, gait trainer, stander, walker, and other (bicycle, stroller, crutches). 49% used a feeding tube, 47% used orthotics. 27% had a tracheotomy. 23% used a communication device, and 20% used help with secretion management. Types of communication devices used were augmentative and alternative communication (AAC) devices, picture books, and sign language. Overall, participants used 0-7 disease management interventions, with mean and median of 2 (SD 1.6). Categorical distribution on the number of interventions is shown in Appendix, Fig. 10.

**Table 4:** Disease management

			Age first used (years)		
Disease management device	N^a^	Disease management device used	N^b^	Mean (SD); Mode	Median (Q1, Q3); Min, Max
		n (%)			
Mobility device	113	81 (71.68)	52	3.5 (5.9); 2	2.0 (1.1, 3.0); 0, 40
NG/JG/G tube	112	55 (49.11)	44	2.9 (3.1); 3	1.8 (1.0, 3.5); 0, 17
Orthotics	106	50 (47.17)	42	2.4 (1.5); 2	2.0 (1.4, 3.0); 1, 8
Tracheotomy	112	30 (26.79)	25	3.6 (1.8); 3	3.0 (2.0, 5.0); 1, 7
Communication device	110	25 (22.73)	20	4.6 (5.1); 3	3.0 (2.5, 5.5); 1, 25
Secretion management	109	22 (20.18)	19	3.1 (2.3); 4	3.0 (1.0, 4.0); 0, 9
CPAP^c^	23	10 (43.48)			
BIPAP^c^	23	9 (39.13)			
Number of interventions used					
Mean (SD); Mode	114	2.2 (1.6); 1			
Median (Q1, Q3); Min, Max		2.0 (1.0, 3.0); 0, 7			

^a^N = number of participants with a valid response (Yes, No) regarding a specific intervention

^b^N = number of participants who used an intervention and provided an age when started

^c^Only participants with a history of sleep apnea were asked about use of CPAP and BIPAP. Age for the start of CPAP and BIPAP was not collected

### Age of symptom onset, lost benchmarks, and disease management

**Fig. 6:**
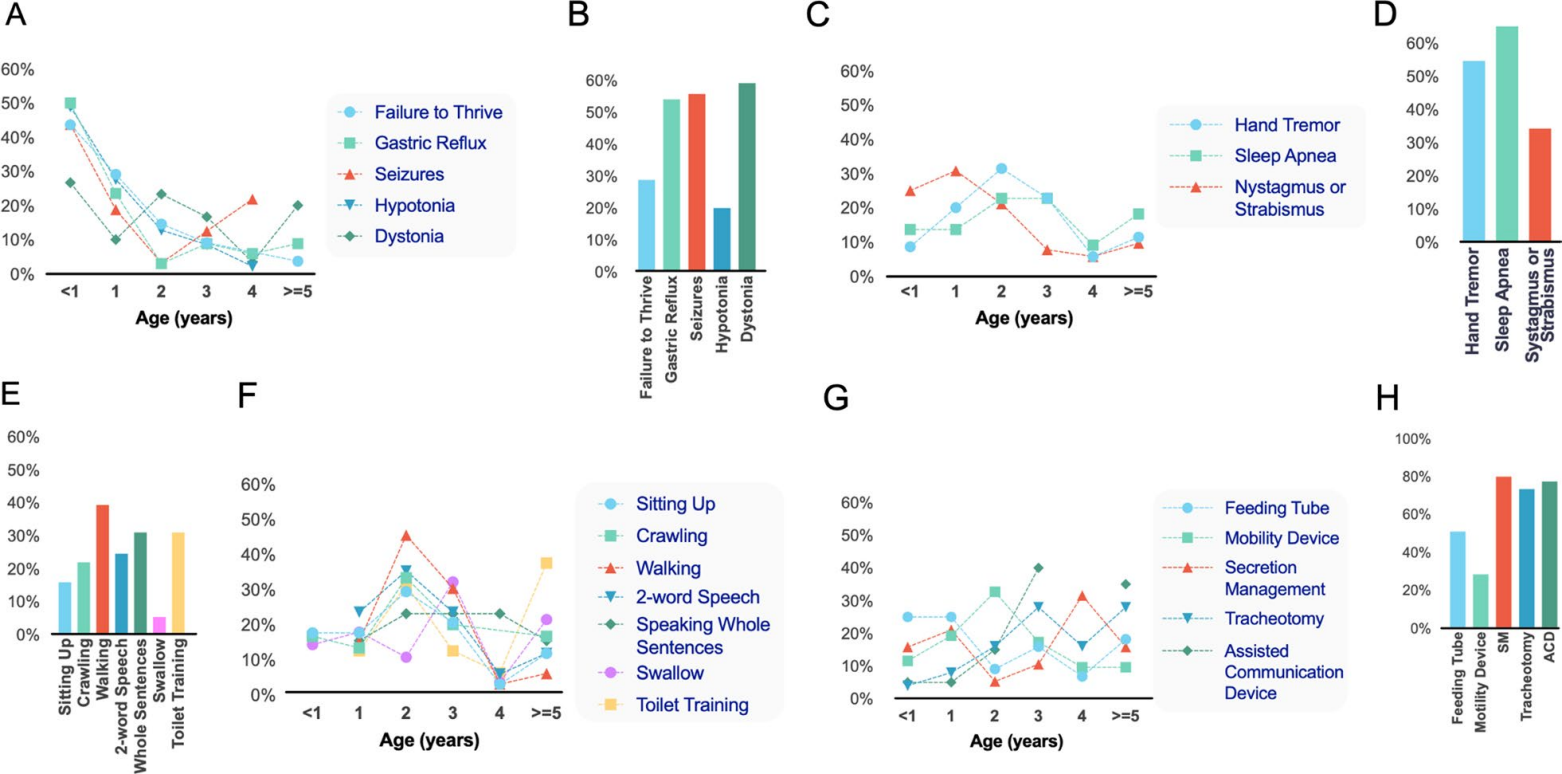
Age of symptom onset, lost benchmarks, and disease management. **A** Distribution of age of onset for major early onset symptoms among patients in whom these symptoms were reported. **B** Fraction of patients who did not present with the symptoms in (**A**). **C** Distribution of age of onset for major later stage onset symptoms. **D** Fraction of patients who did not present with the symptoms in (**C**). **F** Distribution of age of loss of benchmarks. **E** Fraction of patients who never achieved benchmarks in (**F**). (**G**) Distribution of reported age where disease management devices were first utilized among patients. **H** Fraction of patients who did not utilize each respective disease management device. **A**–**H** Patients not included in this analysis if presentation was reported but age of presentation not provided

Relationship between age of symptom onset and lost benchmarks and disease management strategies is shown in Fig. 6.

The distributions of age of symptom onset appear to cluster into two groups: those symptoms most commonly presenting at less than 1 year of age and those most commonly first presenting during the second to third year of life (Fig. 6). Failure to thrive, gastric reflux, seizures, and hypotonia were all most commonly first seen at less than 1 year, with declining rates of first appearance by age. Both hand tremor and sleep apnea are most frequently first reported during the second to third year of life. Dystonia shows a somewhat biphasic distribution, with peaks at both less than 1 year and 2 years of life, while nystagmus or strabismus peaks during the first year.

Milestone loss among patients who achieved a given milestone occurred most frequently during the second year of life. Milestones most commonly lost during the second year include sitting up, crawling, walking, and two-word speech. Toilet training loss peaked during the second year and again after age 5, while swallowing was most commonly lost during the third year.

The distribution of age of first use of disease management interventions varied, but with some clear trends (Fig. 6). Feeding tube use most frequently occurred in younger patients (1 year or less), while mobility device use most commonly occurs first in patients in their second year of life. Tracheotomy and assisted communication device most commonly begins in the third year, while secretion management most commonly begins in the fourth.

### Healthcare utilization

Participants were asked to report the number of emergency room (ER) visits, and days/nights inpatient in the past 3 and 12 months. More than half of respondents reported zero ER or inpatient visits in the past 3 and 12 months (Table 5). The number of ER visits ranged from 1 to 5 over the previous 3 months and 1 to 45 in the previous year for those who reported visiting the ER. Among non-zero responders, the mean number of days in hospital were 12.5 and 26.8 for 3 months and 12 months, respectively. The maximum days in the hospital were 45 days for 3 months and 150 days for 12 months.

**Table 5:** Healthcare utilization

	N^a^	0	1	2	3	> = 4	N^b^	Mean (SD); Mode	Median (Q1, Q3); Min, Max
ER visits in past 3 months	93	72 (77.42)	10 (10.75)	5 (5.38)	4 (4.30)	2 (2.15)	20	1.9 (1.1); 1	1.5 (1.0, 2.5); 1, 5
Nights/days inpatient past 3 months	92	68 (73.91)	1 (1.09)	2 (2.17)	2 (2.17)	19 (20.65)	21	12.5 (12.3); 10	10.0 (4.0, 14.0); 1, 45
ER visits in past 12 months	93	52 (55.91)	19 (20.43)	6 (6.45)	4 (4.30)	12 (12.90)	40	4.0 (7.5); 1	1.5 (1.0, 3.5); 1, 45
Nights/days inpatient past 12 months	91	47 (51.65)	13 (14.29)	2 (2.20)	3 (3.30)	26 (28.57)	40	26.8 (33.6); 1	0.0 (1.0, 59.0); 1, 150

^a^N = number of participants with a valid response regarding a specific type of healthcare utilization

^b^N = number of participants who had response 1,2,3 to a specific type of healthcare utilization or a response of >  = 4 and provided a specific value

### Caregiver burden

**Fig. 7:**
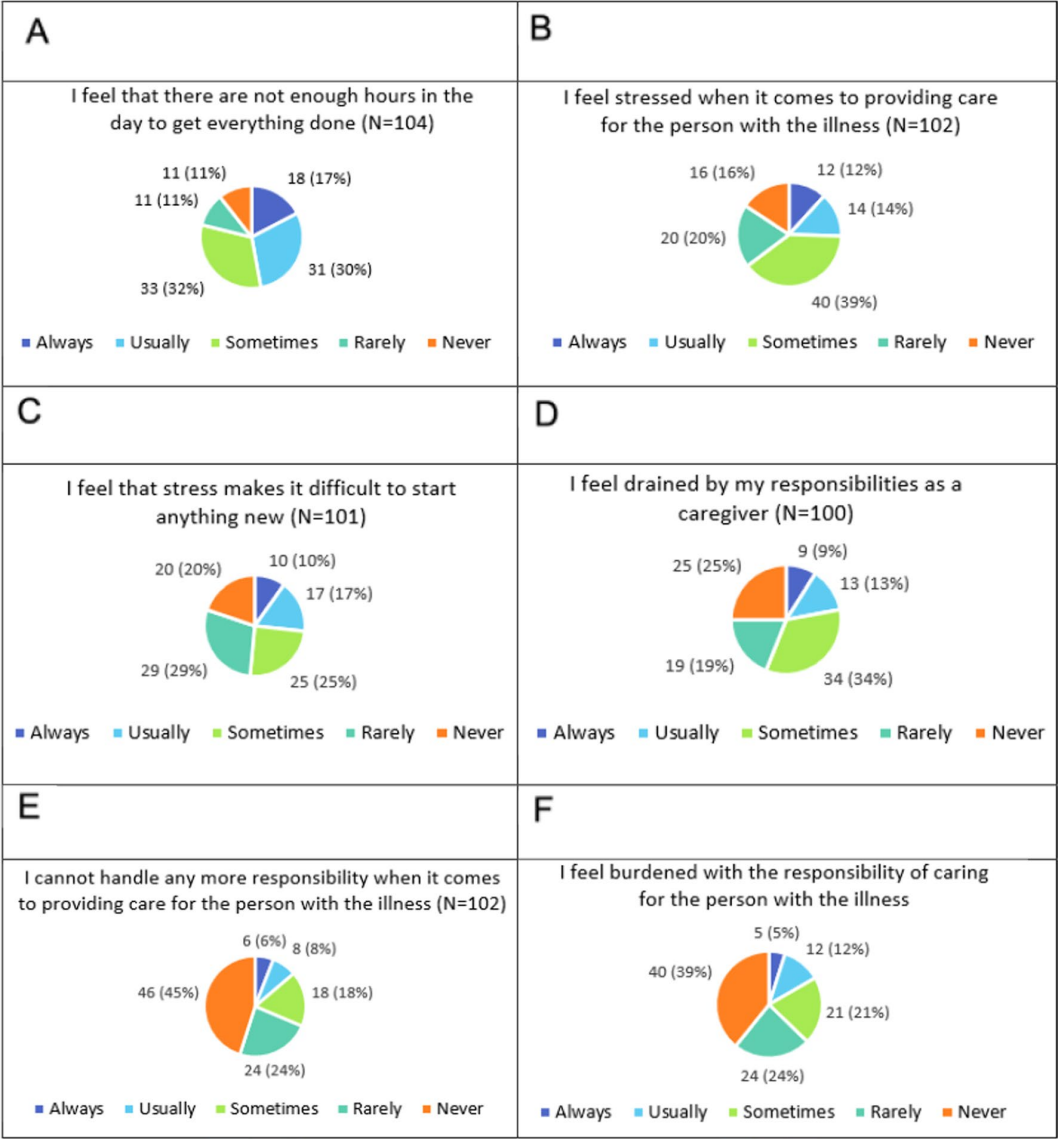
Caregiver burden

Among caregivers, 32% reported sometimes feeling that there are not enough hours in the day to get everything done; 17% always feel this way, and 30% usually feel this way (Fig. 7). 39% of caregivers sometimes reported feeling stressed when it comes to providing care for the person with the illness, with 12% and 14% feeling this way always and usually, respectively. Over half (52%) of caregivers reported feelings that the stress made it difficult to start anything new. 34% reported sometimes feeling drained by their responsibilities as a caregiver, with 9% and 13% feeling this way always and usually, respectively; the remaining 44% of caregivers reported rarely or never feeling drained combined. 18% sometimes feel that they cannot handle any more responsibility when it comes to providing care; only 6% and 8%, respectively, reported feeling this way always and usually. 24% and 45%, respectively, reported rarely or never feeling this way. 21% reported sometimes feeling burdened by providing care, only 5% and 12% reported feeling burdened always and usually, with 24% and 39%, respectively, reported rarely or never feeling burdened.

#### Quality of life

**Fig. 8:**
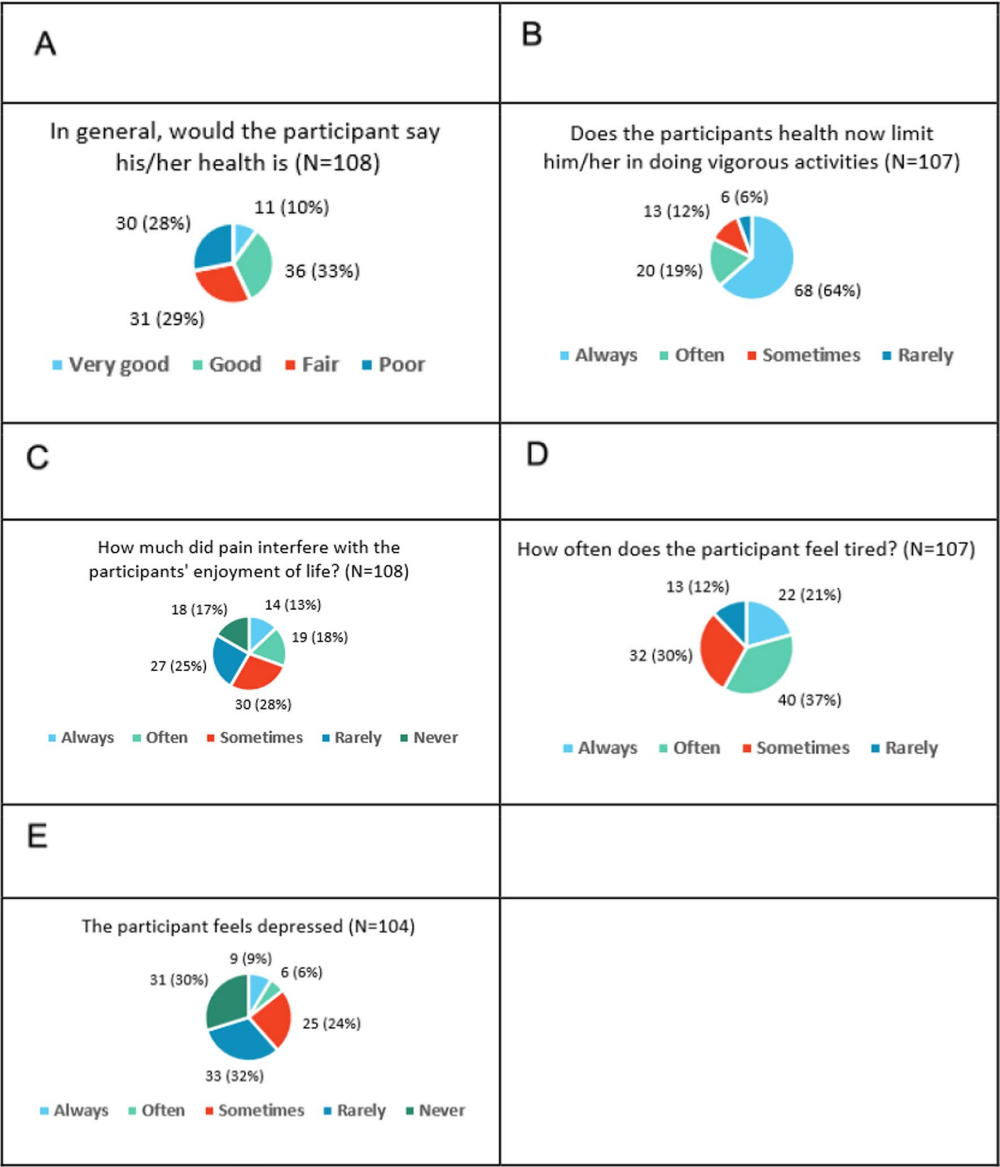
Quality of life

**Fig. 9:**
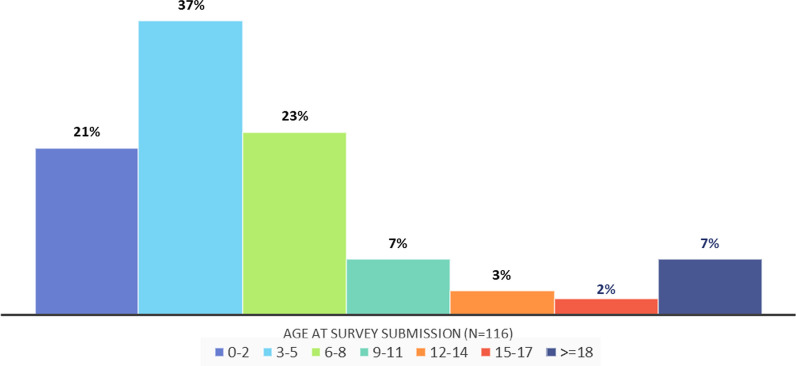
Age at survey submission (years)

**Fig. 10:**
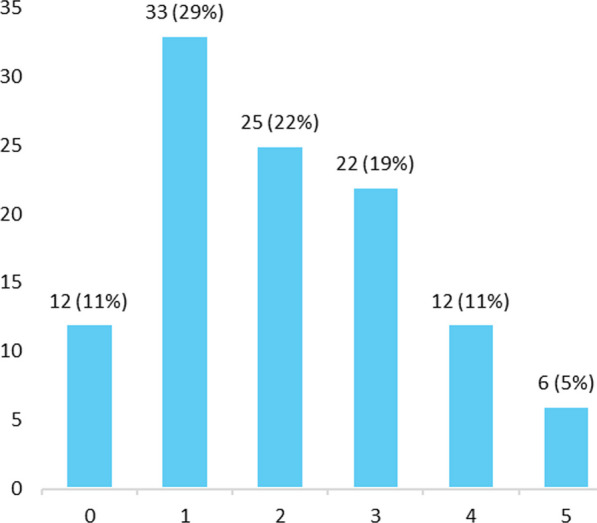
Number of interventions (N^a^ = 114). N = number of participants who responded to questions about interventions

33% reported that participants would rate their health as good. 29% and 28% responded fair and poor, respectively, 10% reported very good (Fig. 8). Majority (64%) reported that a participant's health always limits him/her in doing vigorous activities. Regarding pain, 13% responded that pain always interferes with a participant's quality of life, the largest category was sometimes (28%). 37% reported that the participant often feels tired; nobody responded “never” to this question. Regarding feeling depressed, the largest category was rarely (32%), closely followed by never (30%).

## Discussion

Data collected from a global patient registry for individuals with Leigh syndrome is presented. The authors did not find prior studies on Leigh syndrome reporting outcomes described above such as loss of milestones, specialists seen, disease management, or healthcare utilization, making this the first publication of such results.

Although the registry and Cure Mito Foundation are US-based, nearly 70% of participants reside outside of the US, spanning 8 geographic regions, and 25 countries. Of note, this geographical diversity likely represents the first truly global registry for any mitochondrial disease, a product of the broad-reaching recruitment effort through social media platforms and directly via international Cure Mito partner families. Cure Mito builds connections with patient families from around the world by empowering patients to unite (regardless of language and cultural barriers) to advance research in the Leigh syndrome community. This was initially accomplished with the assistance of international partner families translating the surveys into their own language, being mindful of cultural sensitivity for participants. Subsequent registry enrollment included surveys that were already translated into various languages (Spanish, Portuguese).

Time to diagnosis of Leigh syndrome reported by the participants in the current study is significantly shorter than previously reported in other research on mitochondrial disease overall. A study analyzing data from 210 Rare Diseases Clinical Research Network Contact Registry participants, with self-reported diagnosis of mitochondrial disease found participants saw an average of 8.1 clinicians and over half received 1 or more non-mitochondrial diagnoses before their final mitochondrial diagnosis. [11] Another study, conducted in the Department of Neurogenetics, Royal North Shore Hospital, UK, on 68 participants, found that the mean time to diagnosis was 6.2 years. [12] However, our study focuses on Leigh syndrome specifically, rather than on all mitochondrial disorders. Furthermore, a study of 130 patients with Leigh syndrome by Sofou and colleagues demonstrated a median elapsed time from disease onset to diagnostic testing was 0.9 years (interquartile range Q25-Q75: 0.2–3.1 years). [13] This is similar to our results, and underscores the fact that findings for mitochondrial disorders as a group may not necessarily be generalized for individual mitochondrial diseases. Possibly, as Leigh syndrome is a very severe disease and is much harder to overlook or misdiagnose compared to more mild types of mitochondrial disease, the time to diagnosis is shorter. Twenty-four (24) distinct genetic loci are represented by the patients in this registry to date, though currently there is a significantly higher number of patients carrying mutations in SURF1. This is likely the result of a significant number of participants being recruited from the Cure Mito Foundation, which originally began as the Cure SURF1 Foundation. While there would undoubtedly be some distortion of normal distribution of clinical data of Leigh syndrome populations due to the higher number of SURF-1 related Leigh syndrome entries, this is expected to diminish as the size and genetic diversity of the registry increases. When the registry size allows, further analyses based on genetic loci, and eventually specific genetic lesions, is likely to offer additional insights into the clinical presentation of Leigh syndrome.

Leigh syndrome is typically described as a severe neurological disorder, which is reflected in the high number of registry participants who see neurologists (78%) and therapists for neurodevelopmental disorders. However, the wide variety and quantity (1–30) of specialists seen by participants reflects the severity and multi-system nature of Leigh syndrome. A lot can be learned from the list of other specialists reported by participants including pulmonologists, cardiologists, and gastroenterologists (among others). It is important for general practice clinicians to look beyond neurodevelopmental concerns and consider referrals to other specialists if appropriate. These percentages also underscore the healthcare burden, financial impact, and imposition on quality of life that multiple specialty visits may have on the individual with Leigh syndrome and their caregiver.

It is important to underscore that the number of specialists seen is significantly affected by the country where the participant lives and the healthcare system as well as standard of living in that country. For example, one of the international participants shared with the Cure Mito Foundation, that their child is bed-bound and unable to ever leave their house due to not having any means of transportation with a wheelchair. The child is occasionally visited at home by their primary care provider. The neurologist sometimes adjusts seizure medications over the phone. Therefore, the child sees only one specialist, but that speaks to the family’s living situation, rather than the child's health.

The first concerns expressed by participants varied but were overwhelmingly connected to developmental delay or regression (68%). A majority, 56%, of participants have never achieved at least one milestone (40% of participants never walked). This again highlights the importance of specialist referral and testing by pediatricians for children losing milestones or experiencing developmental delays.

However, only 16% of participants reported that their child’s health care provider raised a concern. This discordance underscores the importance of swift referral to appropriate specialists and genetic testing to confirm the diagnosis. This is also evidenced by the fact that most of the participants see neurologists and a variety of therapists.

Given the involvement of multiple systems in the disease profile, management of Leigh syndrome requires multimodal therapies to achieve patient outcomes and improve quality of life. Although no curative treatment exists, therapies and devices that target neurological symptoms are commonly used for symptomatic care. Interventions such as mobility devices, feeding tubes including nasogastric, gastro-jejunal, and gastric tubes, orthotics, tracheotomy, communication devices, and secretion management were ranked highly utilized by registry participants. The reported number of medical interventions used ranged from a minimum of no devices up to seven devices. Although not captured in the initial results from the Cure Mito registry, pharmacotherapies have been proposed to be integral for managing Leigh syndrome. Medications, specifically, nutraceuticals with antioxidant properties or that function in the biochemical pathways that result in Leigh syndrome are frequently suggested as possible therapies, although clinical evidence is controversial. Examples of nutraceuticals include Coenzyme Q10, L-carnitine, α-lipoic acid, creatine-monohydrate, biotin, thiamine, and riboflavin [14–16].

The most common symptoms first reported within the first year are in line with previously reported data [17]. Our data demonstrates that there is a correlation with symptoms of failure to thrive, gastric reflux, seizures and hypotonia, more commonly reported earlier in life and increasing child age. These suggest that Leigh syndrome presents with early onset of disease in infancy and emphasis on resolving feeding issues, providing nutritional alternatives and seizure management are important. In addition, recognition of these early clinical features may aid earlier diagnosis and disease management. Interestingly, the higher prevalence of dystonia and movement disorders is observed above the age of 1 year and remains relatively consistent with age suggesting brain stem and basal ganglia involvement and supports the assessment of early identification by MRI. Of the patients who reported a history of regression or loss of acquired skills, the majority had common symptoms of abnormal development, including verbal delay (2 word speech), gross motor delay (sitting, crawling, walking) and social delay (toilet training). This data correlates with our findings of disease management and the early requirement for devices to assist daily living. The commonly reported first symptoms of failure to thrive and gastric reflux in infancy correlates with the reported use of a feeding tube in patients less than 1 year old. As the child's age increases, the need for assisted motor and speech devices correlates with reported data of gross motor and verbal delays.

As our registry grows, we anticipate that that our improved understanding of the ages and order of symptom onset in Leigh syndrome will lead to earlier diagnosis and improved opportunities for clinical management. An earlier consideration of Leigh syndrome as a differential diagnosis in pediatric patients displaying any of the early symptoms shown in Fig. 6 would be expected to contribute to an accelerated diagnosis in Leigh syndrome patients. While effective clinical interventions are not yet available, exciting recent preclinical studies have raised the possibility that treatments may soon be available [18, 19]. Any progress in accelerating diagnosis will undoubtedly contribute to improved responses to therapy, as it is anticipated that neurodegenerative changes will be easier to prevent than reverse. Earlier diagnosis may also speed drug development, as sicker patients have a narrower therapeutic window for intervention. Progress toward statistically rigorous natural history of disease in Leigh syndrome will also provide improved control data against which to judge the efficacy of any experimental intervention.

Healthcare system utilization is significant but varies widely across the respondents, as measured by number of ER visits, and days spent in inpatient care. The majority of patients did not visit the ER. However, among those who did, the number of visits ranged from 1 to 45 over the previous twelve months. Among non-zero responders, the mean number of days in the hospital were 12.5 and 26.8 for 3 months and 12 months, respectively. Notably, one respondent indicated 150 days of inpatient care. Overall, the data suggest that for patients whose Leigh syndrome is well managed and stable, healthcare utilization is relatively low. However, when patients do experience symptoms they require significantly more medical care.

The Everylife Foundation completed a study of the economic burden of rare diseases in the US in 2019 [20]. This study concluded that the total excess direct medical cost associated with the 379 diseases studied is $418 billion, which translates to $26,887 excess direct medical costs per person with a rare disease. In fact, the costs are even higher for children, averaging $32,037 excess medical costs per child.

According to the study, the estimated total indirect and non-medical cost of rare disease is $64 billion for children (age less than 18 years). For many Leigh syndrome families, one of the spouses must give up their employment to take care of the affected child. In addition, the costs of absenteeism for the caregivers surpass those for people with the rare disease.

Leigh syndrome was not represented in the 379 rare diseases surveyed in the Everylife study. It is also difficult to extrapolate these cost estimates to the 25 countries represented in the Leigh syndrome global registry due to differing healthcare systems and medical practice. However, the low median age of patients in the registry suggests that the per person cost of healthcare utilization for Leigh syndrome is likely to be higher than for diseases in older populations.

It is interesting to note that despite reported limitations related to pain and energy, the majority responded that participants rated their own health as good, and the majority never or rarely feel depressed. Although this finding may seem counterintuitive, it is supported by other research. A study of 115 children with severe disabilities and their parents, found that parents describe their children's health related quality of life (HRQoL) significantly higher than physicians do and express more optimism regarding their child’s future compared to physicians [21].

One way of bridging the disparity between physician and patient/family is for physicians to go beyond solely having a medical outlook and proactively engage patients in discussions about their QofL. Authors of the article, “Asset-Based Health Care for Children With Severe Neurologic Impairment” explain that parents of children with severe neurological impairments want their clinical team to see their child as they do, including knowing what brings the child and the family joy, hope, and meaning [22]. They suggest that clinicians have conversations with the family that go beyond the medical diagnosis and proactively ask about the child’s QofL as well as what are the hopes of the parents for their child’s future.

As illustrated by the current study, the majority of caregivers feel stressed when it comes to providing care to the person with the illness. A significant percentage (over a third) at least sometimes feel drained by their responsibilities as a caregiver. However, the majority reported never or rarely feeling burdened with the responsibility of caring for the person with the illness. In our opinion, this could be explained by the continuum of hope and love parents feel for their children. During the shared journey, parents provide care for their children out of love and do not view them as a burden. A cross-sectional study on 249 caregivers of children with chronic diseases revealed that “those caregivers who were responsible for caregiving for a longer period, experienced lower caregiving burden in isolation and disappointment sub-scales. It seems that they have found approaches to coping with their responsibilities and caregiving-induced limitations over time” [23].

Although our study showed that caregivers are resilient and those living with Leigh syndrome have a positive attitude, we know that Leigh syndrome is a severe progressive disease, and it is important to have support resources available. It is also possible that those families who are coping better have joined our registry, while for those having a harder time, it may have been too difficult or time-consuming, and we didn’t capture their responses.

## Limitations

One of the main limitations of this study is that most common genetic mutations of the participants may not accurately represent the prevalence and genetic diversity in the general population overall or by country. One of the reasons for this is that Cure Mito Foundation was formerly named Cure SURF1 Foundation and therefore has been primarily connected with SURF1 families until recently. This should change over time as Cure Mito continues to expand. Additionally, as the majority of patient recruitment has been done by patient families, countries and mutations represented reflect in a large part more active families or patient communities. Moving forward, the global registry will continue to leverage the dedication of the partner families and also broaden recruitment efforts. Support of mitochondrial disease clinicians in sharing the registry with patients would be important in order to increase enrollment and get a more even distribution of mutations.

It was observed that certain survey prompts were misinterpreted by participants, given the diversity of countries and cultures. For example, race is not a global concept, and the Cure Mito Foundation has discovered that participants in some countries did not understand how to respond. After clarifying, some have updated their responses, but not all did, which likely contributes to a high number of unknown responses for race. Other questions may have also been understood slightly differently due to the variety of languages and countries represented. Translation of surveys into multiple languages should mitigate this concern going forward.

Other responses, such as specialists seen and healthcare utilization, are also affected by the country where the participant lives and the living standard and healthcare system in that country. As described in the discussion above, some international patients are completely house-bound due to not having any way to travel in a wheelchair. An analysis by country may be important to undertake in the future.

Another limitation is that genetic diagnosis was typed in by the participants themselves. Participants had an option to upload their genetic reports and many did; however, the reports have not been used to verify each participant’s mutation. Additionally, all data is reported by patients or their caregivers and information entered has not been verified by medical professionals.

## Future efforts

Cure Mito Foundation plans to continue registry recruitment efforts from around the world, expanding the number of languages available for the survey and further clarifying intent for some culturally sensitive questions. Additional subgroup analysis may be undertaken, such as analysis by age group, country, genetic mutation, or other relevant categories. Publication of additional results and findings, as well as additional subgroup analysis, in various communities is forthcoming.

As the registry expands, Cure Mito Foundation plans to continue to keep participants engaged by regularly sharing results through posters, papers, newsletters, webinars, and other methods and through collaborations with patients and patient advocacy organizations. Additionally, Cure Mito Foundation empowers patients by encouraging them to ask questions, share feedback and suggestions, and by being available and open for communication. Support of the medical community is also important and would greatly assist in expanding the registry.

To continue to build partnerships across the entire ecosystem of rare diseases, Cure Mito Foundation is working with the CoRDS registry to implement a Global Unique Identifier (GUID), making it possible to identify data for the same individuals and different datasets and combine their data for further research. This is only possible if GUID is defined the same way in multiple databases. CoRDS registry will be creating GUID, using the NIH GUID generator [24, 25].

Furthermore, since 2021 Cure Mito Foundation has been partnering with Critical Path Institute (C-path) to significantly promote data sharing and accelerate Leigh syndrome and other rare mitochondrial disease data incorporation into C-Path’s Rare Disease Cures Accelerator-Data and Analytics Platform (RDCA-DAP®) [26]. RDCA-DAP platform is funded by the Food and Drug Administration (FDA) and provides a centralized and standardized infrastructure to support and accelerate rare disease characterization targeted to accelerate clinical drug development [27]. Leigh syndrome patient registry data has already been incorporated into RDCA-DAP and Cure Mito Foundation and C-path continue to collaborate to bring other mitochondrial disease stakeholders into the collaboration [28].

Cure Mito Foundation is also collaborating with Sumptuous Data Sciences, LLC on converting patient registry data into Clinical Data Interchange Standards Consortium (CDISC) standard, which is a regulatory standard required by the FDA and PMDA [29]. This will make the data useful in order to interact with regulatory agencies and possibly support a regulatory submission. To our knowledge, this is the first patient registry in mitochondrial disease to undergo CDISC conversion.

Cure Mito Foundation continues to be committed to collaboration, partnerships, and making sure the data is as accessible and available as possible. Those researchers and industry partners who would like to collaborate, receive data, share research or clinical trial opportunities with the patient community are strongly encouraged to reach out to the Cure Mito Foundation.

## Conclusions

This study describes the first publication on a global Leigh syndrome patient registry with a variety of outcomes collected from participants that can serve as a basis for future natural history studies on Leigh syndrome. Results describe a high burden of the disease, as evidenced by the number of specialists seen, disease management interventions, and healthcare utilization. Time to diagnosis is relatively short compared to prior research on mitochondrial disease overall and underscores the importance of differentiating between mitochondrial disease overall and individual mitochondrial disorders. Despite being overall resilient, caregivers report significant amounts of stress, and finding ways to support caregivers is important. Participants in general are described as having a good quality of life, despite the limitations that they are facing. Future efforts with the registry will include continued collaboration with researchers and industry, continued publication of the results, possible subgroup data analysis, learning new insights that will come out through integration of the data into the RDCA-DAP platform by C-PATH, and leveraging CDISC conversion of the data in order to advance use of the data with the regulatory agencies. Increased efforts will be made to recruit more participants into the registry. This will be done through continued efforts by the Cure Mito Foundation and international patient families, as well as raising awareness of the registry with the mitochondrial disease clinicians worldwide. Our belief and hope is that through all these efforts, our patient registry will play a vital role in making treatments for Leigh syndrome approved and available to patients.

## Data Availability

Raw data from Leigh syndrome patient registry can be obtained by making a request at: https://research.sanfordhealth.org/rare-disease-registry/researchers. The datasets used and/or analyzed during the current study are available from the corresponding author on reasonable request.

## Appendix

See Figs. 9, 10 and Tables 6, 7, 8, 9.

**Table 6:** Participants Enrollment

Enrolled	182
Excluded	66
Exclusion reason	
Did not enter any data	27
Did not complete one or both surveys	18
Diagnosis not confirmed by genetic testing	7
Genetic testing information missing	3
Gene not reported	9
Confirmed to be asymptomatic carrier	2
Included in analysis	116

**Table 7:** Participant country by geographic region (N = 116)

Region	Country	n (%)
Eastern Europe	Russia	32 (27.59)
	Belarus	5 (4.31)
	Romania	3 (2.59)
	Ukraine	2 (1.72)
	Bulgaria	1 (0.86)
	Czech Republic	1 (0.86)
	Hungary	1 (0.86)
	Poland	1 (0.86)
North America	United States of America (USA)	36 (31.03)
Western Europe	Germany	3 (2.59)
	Italy	3 (2.59)
	United Kingdom	3 (2.59)
	Austria	2 (1.72)
	France	2 (1.72)
	Spain	2 (1.72)
	Greece	1 (0.86)
Latin America and the Caribbean	Argentina	3 (2.59)
	Brazil	3 (2.59)
	Mexico	2 (1.72)
	Ecuador	1 (0.86)
Near East	Israel	3 (2.59)
Central Asia	Kazakhstan	1 (0.86)
	Turkmenistan	1 (0.86)
Oceania	Australia	2 (1.72)
Asia	India	1 (0.86)
Unknown	Not reported	1 (0.86)

**Table 8:** Type of genetic testing used to determine diagnosis (N = 116)

Testing type	n (%)
Whole Exome sequencing	28 (24.14)
Mitochondrial DNA sequencing	26 (22.41)
Whole genome sequencing	22 (18.97)
Don't know	11 (9.48)
Other	11 (9.48)
Gene panel	8 (6.90)
Targeted gene analysis	8 (6.90)
Not reported	2 (1.72)

**Table 9:** Specialists seen (N<Superscript>a</Superscript> = 105)

Specialist type	n (%)
Neurologist	82 (78.10)
Physical therapist	67 (63.81)
Geneticist	55 (52.38)
Cardiologist	51 (48.57)
Occupational therapist	50 (47.62)
Nutritionist	48 (45.71)
Speech therapist	46 (43.81)
Gastroenterologist	32 (30.48)
Pulmonologist	30 (28.57)
Not reported	6 (5.71)
Other	4 (3.81)

^a^Living patients only
